# Radiosurgery in Treatment of Ventricular Tachycardia – Initial Experience Within the Polish SMART-VT Trial

**DOI:** 10.3389/fcvm.2022.874661

**Published:** 2022-04-18

**Authors:** Radosław Kurzelowski, Tomasz Latusek, Marcin Miszczyk, Tomasz Jadczyk, Jacek Bednarek, Mateusz Sajdok, Krzysztof S. Gołba, Wojciech Wojakowski, Krystian Wita, Rafał Gardas, Łukasz Dolla, Adam Bekman, Aleksandra Grza̧dziel, Sławomir Blamek

**Affiliations:** ^1^Department of Cardiology and Structural Heart Diseases, Medical University of Silesia, Katowice, Poland; ^2^Department of Radiotherapy, Maria Skłodowska-Curie National Research Institute of Oncology, Gliwice, Poland; ^3^Interventional Cardiac Electrophysiology Group, International Clinical Research Center, St. Anne’s University Hospital Brno, Brno, Czechia; ^4^Department of Electrocardiology and Heart Failure, Medical University of Silesia, Katowice, Poland; ^5^Department of Electrocardiology, Prof. Leszek Giec Upper-Silesian Medical Centre of the Medical University of Silesia, Katowice, Poland; ^6^Department of Electrocardiology, John Paul II Hospital, Kraków, Poland; ^7^First Department of Cardiology, Medical University of Silesia, Katowice, Poland; ^8^Department of Radiotherapy Planning, Maria Skłodowska-Curie National Research Institute of Oncology, Gliwice, Poland; ^9^Department of Medical Physics, Maria Skłodowska-Curie National Research Institute of Oncology, Gliwice, Poland

**Keywords:** radioablation, electrical storm, structural heart disease, arrhythmia-stereotactic body radiotherapy, ventricular tachycardia

## Abstract

**Background:**

Stereotactic Arrhythmia Radioablation (STAR) is an emerging treatment modality for patients with sustained ventricular tachycardia (VT) and refractory to treatment with drugs and radiofrequency catheter ablation (RFA). It is believed that up to 12–17% of patients experience recurrence of VT within 1 year of follow-up; thus, novel therapeutic options are needed. The aim of this article is to present initial experience within a novel treatment modality for VT.

**Case Summary:**

Two patients with a medical history of coronary artery disease and heart failure with reduced left ventricle (LV) ejection fraction, after implantation of cardioverter-defibrillator (ICD) and previous unsuccessful RFAs owing to sustained VT were admitted to the cardiology department due to recurrence of sustained VT episodes. With electroanatomical mapping (EAM), the VT substrate in LV has been confirmed and specified. In order to determine the target volume for radioablation, contrast-enhanced computed tomography was performed and the arrhythmia substrate was contoured using EAM data. Using the Volumetric Modulated Arc Therapy technique and three 6 MeV flattening filter-free photon beam fields, a single dose of 25 Gy was delivered to the target volume structure located in the apex and anterior apical segments of LV in the first patient and in the apex, anterolateral and inferior apical segments of the second patient. In both cases, volumes of the target structures were comparable. Interrogation of the implanted ICD at follow-up visits throughout 6 months after the treatment revealed no VT episodes in the first patient and sudden periprocedural increase in VT burden with a subsequent gradual decrease of ventricular arrhythmia to only two non-sustained episodes at the end of the follow-up period in case of the second patient. A significant reduction in premature ventricular contractions burden was observed compared to the pre-treatment period. No noticeable deterioration in LV function was noted, nor any adverse effects of radiosurgery associated with the implanted device.

**Conclusion:**

The early response to STAR can be unpredictable and probably does not reflect the final outcome of irradiation. Close monitoring of patients, especially in the early period after irradiation is crucial to properly handle potentially harmful early reactions to STAR.

## Introduction

Stereotactic Arrhythmia Radioablation (STAR) is an emerging treatment modality for patients with persistent ventricular tachycardia (VT), a potentially life-threatening disorder caused by malfunctioning electrical conduction of the myocardium often associated with structural heart disease (1). The therapeutic options include implantable cardioverter-defibrillator (ICD), antiarrhythmic medications, and percutaneous radiofrequency catheter ablation (RFA) of the arrhythmic substrate (2). The ICD improves survival rate at the expense of a patient’s quality of life and increases the possibility of exacerbating the underlying cardiac failure. Although capable of reducing the VT burden, antiarrhythmic medications are often associated with considerable toxicity. Finally, RFA can permanently terminate VTs; however, up to 12–17% of patients experience recurrence as soon as at 1 year of follow-up (3), indicating a pressing need for novel therapeutic options.

To no surprise, the first case reports on STAR (4–6) and encouraging results of the phase I/II study by Robinson et al. (7) have met with avid interest, resulting in multiple centers adopting the method as compassionate treatment and within new prospective clinical trials (8). One of them is the Polish trial SMART-VT (ClinicalTrials.gov Identifier: NCT04642963) (9). It was launched on September 11, 2020, and aims to evaluate treatment safety, as described in detail in the trial protocol (10). Briefly, the inclusion criteria are: structural heart disease and implanted cardioverter-defibrillator (ICD), clinically significant arrhythmia with at least 3 VT episodes per month despite adequate pharmacological treatment, at least one episode of monomorphic VT registered during the electrophysiological study, recurrent VT despite at least one prior catheter ablation and adequate pharmacotherapy or contraindications to catheter ablation and/or pharmacotherapy, ability to understand and will to sign a written informed consent document. Here we present the cases of the first two patients treated in Poland within the SMART-VT trial.

## Case Report

### Cases Presentation

#### First Patient

A 69-year-old man with a medical history of coronary artery disease, heart failure with reduced ejection fraction (left ventricle ejection fraction of 22% and New York Heart Association class II), after ICD implantation and previous unsuccessful RFAs was admitted to the cardiology department in December 2020, due to sustained, clinically significant VT. Despite previous conventional RFAs, the VT substrate persisted and led to an electrical storm with recurrent episodes of ventricular tachycardia, which demanded hospitalization approximately a month before the admission to our hospital. The ICD interrogation revealed 14 episodes of VT (7 episodes of non-sustained VT and eight episodes of sustained VT). Sustained episodes were treated successfully with anti-tachycardia pacing (ATP). Furthermore, the device interrogation disclosed 76 episodes of VT with 302 ATPs since device implantation. Considering the limited remaining options of therapy and ineffectiveness of the past RFAs, the patient was enrolled in the SMART-VT trial.

#### Second Patient

A 72-year-old patient with a history of coronary artery disease and heart failure with reduced ejection fraction (left ventricle ejection fraction of 20% and New York Heart Association class III) was admitted to the cardiology department in December 2020 due to an electrical storm with two adequate, high-voltage interventions. The patient had a history of ICD implantation (with a subsequent upgrade to cardiac resynchronization therapy) and underwent several RFAs procedures in the past. Prior to the STAR procedure, the CRT-D interrogation was performed, confirming five high-voltage interventions. The patient was also enrolled in the SMART-VT trial.

### Treatment

**FIGURE 1:**
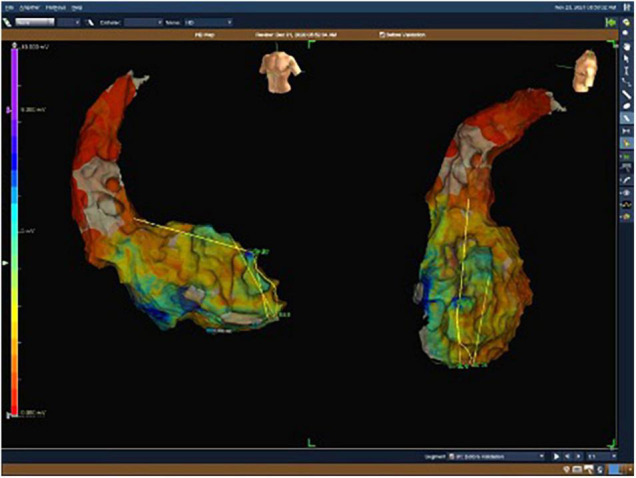
Electroanatomical map with unipolar (UV) voltage of the first patient. UV range from 0.05 mV (red color) to 8.3 mV (purple color).

**FIGURE 2:**
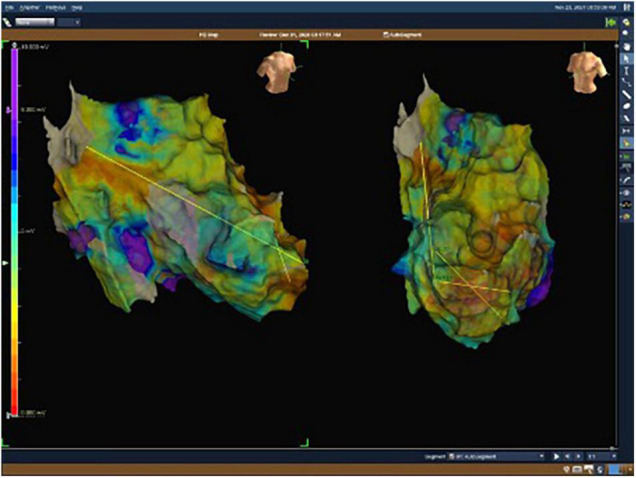
Electroanatomical map with unipolar (UV) voltage of the second patient. UV range from 0.05 mV (red color) to 8.3 mV (purple color).

**FIGURE 3:**
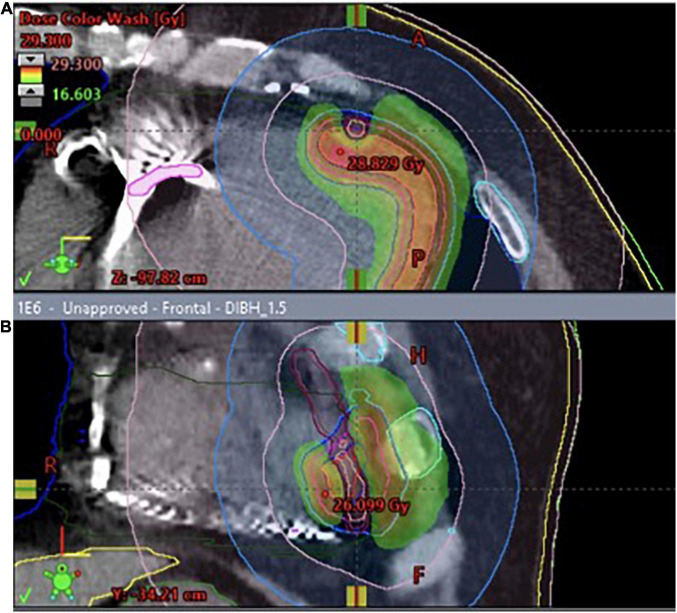
**(A)** 3D visualization of the treatment plan with three coplanar dynamic arcs (VMAT technique), spatial reconstruction of contoured structures and DRRs (Digitally Reconstructed Radiograms) used for preliminary positioning of the patient. The final adjustment was performed using cone-beam CT (CBCT) superimposed on the planning CT. **(B)** The dose distribution was optimized to cover the planning target volume while accounting for organs-at-risk such as coronary arteries. Red and orange color represent higher dose, green- lower.

After excluding reversible causes of VT, both patients underwent 3D electroanatomic mapping (EAM) using The EnSite Precision™ Cardiac Mapping System by Abbott. Based on the obtained electrophysiological map (Figures 1, 2), data from previous RFAs and computed tomography (CT), the target volume for radioablation was specified and transferred to the Varian ECLIPSE™ treatment planning system. The target volume contouring was performed on the spot, using a remote access workstation, through indirect comparison of EAM data and contrast-enhanced CT fused with Deep Inspiration Breath Hold (DIBH) treatment planning CT. The organs-at-risk (OARs) and derivative structures were prepared according to the study protocol (10). Using Volumetric Modulated Arc Therapy (VMAT) technique, three 6 MeV flattening filter-free photon beam fields, DIBH respiratory motion management, a dose of 25 Gy was delivered to the planning target volume (PTV) (Figure 3A). It was localized in the apex and anterior apical segments of LV in the first patient and in the second patient’s apex, anterolateral and inferior apical segments. In both cases, volumes of the target structures were similar and equaled 56.37 and 56.72 cm^3^, respectively. According to the safety-first paradigm, the dose was reduced to account for OARs such as coronary arteries (Figure 3B). Dose constraints for OARs and dose to target are presented in table below (Table 1). Most of them are based on values for thoracic stereotactic radiotherapy, however, dose constraints for coronary arteries have been extrapolated form available data with principle of maximum safety (10). Each RT fraction is supervised by a cardiologist. In accordance with national recommendations of the Heart Rhythm Section of the Polish Cardiac Society and the Polish Society of Radiation Oncology several safety procedures are used: continuous audiovisual contact with the patient, ECG monitoring, pulse oximetry, and capillary pulse wave recording. An access to an external defibrillator and also to external stimulation options and portable programmer is provided. During RT, it is recommended to temporarily switch off ventricular tachycardia/ventricular fibrillation detection (11).

**TABLE 1:** Dose constraints for organs-at-risks (OARs) and dose to target.

OAR	Volume	Volume dose	Point dose*
PTV minus CTV	–	–	31.25 Gy
CTV	<1 cm^3^	32.5 Gy	35 Gy
Spinal cord	<0.35 cm^3^	10 Gy	14 Gy
	<1.2 cm^3^	8 Gy	
Esophagus	<5 cm^3^	11.9 Gy	15.4 Gy
Stomach	<5 cm^3^	17.4 Gy	22 Gy
Duodenum	<5 cm^3^ <10 cm^3^	11.2 Gy 9 Gy	17 Gy
Trachea and main bronchi	<4 cm^3^	17.4 Gy	20.2 Gy
Lungs (together)	<1500 cm^3^	7 Gy	
	<1000 cm^3^	7.6 Gy	
	<37%	8 Gy	
Liver	<700 cm^3^	11 Gy	
Kidneys (together)	<200 cm^3^	9.5 Gy	
Coronary arteries ^	–	–	12 Gy
Ribs	<5 cm^3^	28 Gy	33 Gy
Skin	<10 cm^3^	25.5 Gy	27.5 Gy

*Defined as point dose in < 0.035 cc. ^ Left coronary artery including anterior intraventricular and circumflex, and right coronary artery including posterior descending artery.

The whole radiotherapy session took approximately 35 min, including 13 min of beam-on time, using a C-arm linear accelerator EDGE by Varian. No substantial acute toxicity was observed. Except for mild discomfort associated with the treatment session, both patients remained free of adverse effects until the final discharge from the hospital ward 2 days later.

## Results

### First Patient Follow-Up

**FIGURE 4:**
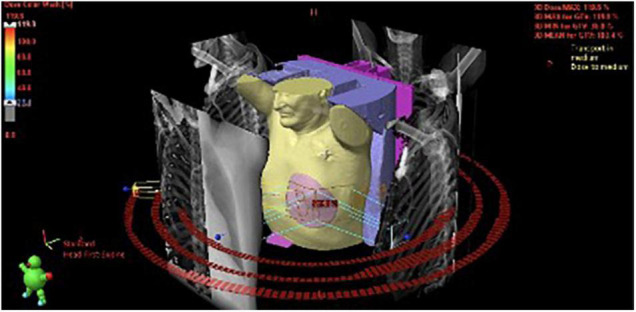
Premature ventricular contractions burden before and after the treatment of the first patient.

Interrogation of the implanted ICD revealed no VT episodes throughout 6 months of follow-up. A significant reduction in premature ventricular contractions burden was observed compared to the pre-treatment period (Figure 4). No noticeable deterioration in LV function was noted, nor any adverse effects of radiosurgery associated with the implanted device. The laboratory tests did not show any myocardial damage both at 3 and 6-months after treatment, and the patient did not report any clinically relevant adverse effects of radiosurgery.

### Second Patient Follow-Up

During 6 months of follow-up since the initial procedure, interrogation of the implanted CRT-D was performed multiple times. In the periprocedural period (4 days after irradiation), 67 episodes of VT (7 episodes of non-sustained VT and 60 episodes of sustained VT) were revealed, treated with two high-voltage interventions and 60 ATPs. The patient was immediately admitted to the hospital with considerate hypokalemia and discharged 2 days later with supplementation of electrolytes, intravenous amiodarone administration and alteration of pharmacotherapy. Three weeks after the STAR procedure, the patient was hospitalized again due to reported chest pain and alleged device intervention. During the hospitalization, the CRT-D was interrogated and no high-voltage interventions nor the history of VT were disclosed. No deterioration of LV function was noted nor laboratory tests changes. The patient was discharged from the ward with the diagnosis of intercostal neuralgia 2 days later. Another hospitalization was required 3 months after STAR due to congestive heart failure exacerbation with pulmonary congestion, pleural effusion, and peripheral pulmonary embolism. The patient received inotropic agents (dopamine, levosimendan), and right pleural thoracentesis was made with the evacuation of transudative fluid. LVEF remained unchanged (EF = 20%). Until the end of the 6-months of observation, an additional two non-sustained VT (nsVT) episodes were recorded. However, the number of VT episodes during this observation period decreased considerably compared to the periprocedural period.

## Discussion

To the best of our knowledge, this article depicts the first two patients treated with STAR in Poland and demonstrates the possibility of stereotactic radiosurgery application for the treatment of VT in patients with post-RFA recurrence.

Eventually, in both patients treatment success can be announced; however, post-STAR courses differed significantly in each case. The initial concept of the treatment was based on the assumption that the biological mechanism of STAR leads to transmural fibrosis in the region of the arrhythmogenic substrate and subsequent cessation of electric signal propagation. The delivery of 25–35 Gy to the target area induces fibrosis within 6 months of irradiation, as shown on animal models (12). However, clinical studies demonstrated that STAR is capable of inducing immediate treatment effect (13, 14). The first patient treated in our study presented an excellent response to STAR treatment with no ventricular arrhythmia recurrence or LV function deterioration during 6 months of follow-up. The STAR effect was rapid, with an evident reduction in premature ventricular contractions (ICD interrogation, electrocardiography) and improvement in the patient’s symptoms (quality of life questionnaire). Such swift response is probably an effect of functional changes in the myocardium after STAR. One of the possible explanations was described by Zhang et al. (13). The authors demonstrated alteration of Notch signaling pathway resulted with upregulation of NaV1.5 and Cx. This may influence the conduction velocity and thus, represent a mechanistic explanation of the observed phenomena. In one patient the manifestation of the physiologic processes was anticipated and expressed by rapid reduction of the VT burden, whereas in the other the severity of arrhythmia surprisingly increased.

This demonstrates the complexity of the myocardial response to irradiation and indicates that the physiological phenomena standing behind the final clinical effect are still largely unknown and require more in-depth evaluation than previously stipulated.

As mentioned above, the treatment course of the second patient was more complex. During the follow-up period, the patient required a few hospitalizations not necessarily related to irradiation, however we cannot exclude congestive heart failure exacerbation, pleural effusion and peripheral pulmonary embolism as adverse events potentially related to STAR (reported according to commonly used terminology criteria for adverse events as Grade 3 in SMART-VT Trial). Nevertheless, the second patient’s LV function remained unchanged during the follow-up. A transient ventricular arrhythmia intensification was observed during his periprocedural period with eventually sustained VT absence at 6 months of follow-up. The second patient’s response to the treatment theoretically could also be a manifestation of transient post-procedural cardiac tissue damage followed by concurrent electrical and structural changes, so initially expressed by a marked dysregulation of the cardiac function but finally leading to the expected clinical result. The post-STAR course differing significantly in each case indicates that an individual approach is required after the procedure and the clinical course of the disease after STAR can be unpredictable.

Stereotactic Arrhythmia Radioablation can be performed using conventional (c-arm) or CyberKnife (CK) accelerators. Both techniques have their advantages and disadvantages. First of all, it is important to be mentioned that the main difference between these two machines is the way in which target volume is irradiated. CK uses multiple small beams (tens-hundreds) to irradiate target volume while Linac accelerator irradiates the whole target volume at the same time. This important technical difference result in significant reduction in the delivery time from 60–70 min for CK to 5–6 min for Linac (without DIBH) (15–18). Both CK and Linac, allow to compensate for the respiratory motion. CK uses the respiratory tracking technique (Synchrony) during the whole respiratory cycle. The accelerator head moves synchronously with the respiratory movement of the body following the correlation curve calculated using the data on spatial position of the internal marker used for target tracking linked with the position of the light markers on the vest used for respiratory motion tracking. It allows for continuous delivery of the beam during the respiratory cycle but still, the target is irradiated part by part with numerous beams which causes the beam-on time to be approximately 35–69 min and total treatment time of 65–99 min according to Wang et al. (17). Conventional linac uses the respiratory gating technique (irradiation during the end-expiratory phase of the breathing cycle, usually between the last 10% of expiration and first 10% of the inspiration phase) or DIBH (irradiation during the deep inspiration breath hold) to manage the respiratory motion. The emission of the beam is interrupted in both cases but the number of beams is small (usually 2–4), the whole target volume is irradiated at the same time and the total treatment times are usually 37–56 min and beam delivery time 5–6 min (17). This numbers are similar with numbers observed in our clinical data.

Comparison of treatment plans created for different techniques by Weidlich et al. have shown larger dose gradients nearby PTV for CK plans, showing its advantage in sparing nearby OAR’s comparing to the Linac-based accelerator which is superior in sparing distant OAR’s.

## Conclusion

An individual approach is required after the STAR procedure, and the clinical course of the disease can be unpredictable. Functional changes could appear relatively early, manifested by swift decrease of VT burden, as well as transient exacerbation of the arrhythmia. The SMART-VT study is ongoing, and the clinical course of the two presented cases clearly indicates that the complete toxicity profile of the STAR can be assessed only within a clinical trial.

## Data Availability Statement

The raw data supporting the conclusions of this article will be made available by the authors, without undue reservation.

## Ethics Statement

The studies involving human participants were reviewed and approved by the Komisja Bioetyczna Narodowego Instytutu Onkologii w Gliwicach. The patients/participants provided their written informed consent to participate in this study.

## Author Contributions

All authors contributed to the article and approved the submitted version.

## Conflict of Interest

The authors declare that the research was conducted in the absence of any commercial or financial relationships that could be construed as a potential conflict of interest.

## Publisher’s Note

All claims expressed in this article are solely those of the authors and do not necessarily represent those of their affiliated organizations, or those of the publisher, the editors and the reviewers. Any product that may be evaluated in this article, or claim that may be made by its manufacturer, is not guaranteed or endorsed by the publisher.
